# A Comparative Analysis on the Social Determinants of COVID-19 Vaccination Coverage in Fragile and Conflict Affected Settings and Non-fragile and Conflict Affected Settings

**DOI:** 10.34172/ijhpm.2022.6830

**Published:** 2022-10-10

**Authors:** Sanjay Pattanshetty, Mantej Pardesi, Nachiket Gudi

**Affiliations:** ^1^Department of Global Health Governance, Prasanna School of Public Health, Manipal Academy of Higher Education, Manipal, India.; ^2^Department of Microeconomics and Public Economics, School of Business and Economics, Maastricht University, Maastricht, The Netherlands.; ^3^Public Health Evidence South Asia, Department of Health Information, Prasanna School of Public Health, Manipal Academy of Higher Education, Manipal, India.

**Keywords:** COVID-19, Equity, Fragile and Conflict, Determinants of Health, Vaccine Coverage

## Abstract

**Background:** The coronavirus disease 2019 (COVID-19) pandemic has coerced various resources of all the countries. While the high-income nations redirected financial and human resources to understand specific determinants of vaccination coverage, fragile and conflict-affected setting (FCS) nations were waiting for global bodies to cater to their ever-growing need for vaccines and other lifesaving drugs. This study aimed to determine various factors influencing vaccine coverage in the FCS context.

**Methods:** World Bank’s classification of FCS states was the primary source for country classification. The study utilized data from various other open sources. The study models cross-country inequities in COVID-19 vaccine coverage and we have employed multi-variate log-linear regressions to understand the relationship between COVID-19 vaccine coverage and cross-country macro-level determinants. The analysis was conducted on two samples, non-FCS Countries and the FCS countries.

**Results:** Socio-economic determinants such as gross domestic product (GDP) per capita, socioeconomic resilience; health system determinants such as density of human resources, government spending on health expenditure; and political determinants such as effective government, more power to regional governments, political stability and absence of violence play a pivotal role in vaccine coverage. We also found that FCS countries with a higher share of people strongly believing in the vaccine effectiveness have a positive association with COVID-19 vaccine coverage.

**Conclusion:** The study confirmed that political factors, government effectiveness and political stability are also important determinants of vaccine coverage. The result further draws attention to few policy implications such as promoting future research to explore the linkages between the perceived equality before the law and individual liberty and its effect on vaccination coverage in the FCS.

## Background

**Key Messages**

** Implications for policy makers**

- The research brings to light a comprehensive list of determinants that influence the coverage of coronavirus disease 2019 (COVID-19) vaccination in fragile and conflict-affected setting (FCS).
- The study informs in prioritization and resource allocation for enhancing the COVID-19 vaccination coverage.
- The study calls for creating an enabling environment in FCS to improve vaccine equity.

** Implications for the public**

Coronavirus disease 2019 (COVID-19) had an unprecedented impact on the lives and livelihood of the people. Globally, there were both pharmaceutical and public health interventions implemented to minimize the direct and in-direct effects of the pandemic. However, the success of the interventions was dependent on the social, political, economic and health system factors. COVID-19 effects could be even more severe due to lack of preparedness and response plans to the pandemic in fragile and conflict-affected setting (FCS). To achieve favorable population health outcomes, it is important to understand and improve the governance in FCS to enhance prevention and control measures such as vaccination which further facilitates in achieving better health outcomes.

The coronavirus disease 2019 (COVID-19) pandemic has disrupted all aspects of contemporary society leading to severe health and economic emergencies throughout the world. Evidence from the Ebola Outbreak in West Africa^1^ and the Democratic Republic of the Congo (2018–2019),^2^ show that indirect health effects can exceed the deaths and morbidity directly attributed to the infectious disease.^3^ Humanitarian crises have been seen to be associated with countries which suffer the most severe impact of COVID-19 pandemic.^4^

 The United Nations Security Council resolution 2565 (2021) recognized “that armed conflicts can exacerbate the COVID-19 pandemic, and inversely the pandemic can exacerbate the adverse humanitarian impact of armed conflicts, as well as exacerbating inequalities.”^5^ Countries classified as fragile are further forced to redirect health resources such as manpower and funds to manage the pandemic. Under the pre-existing strained resources, task shifting of health workers might slow down the ongoing efforts and might reverse the gains observed in health outcomes after decades of investments and efforts.^6,7^ Most of the resources in healthcare sector were re-directed for COVID-19 vaccination campaigns to improve the coverage.

 In the non-fragile and conflict-affected setting (non-FCS), the determinants of access to healthcare services like vaccination is related to availability, affordability and attitude at individual level to avail services.^8^ The situation in the non-FCS countries is different, owing to better access, efforts to curtail hesitancy, and other innovations in vaccine delivery.^9^ However, in a FCS context, disrupted supply chain management of COVID-19 vaccines, war and conflict has an effect on availability and also accessibility. United Nations Security Council resolution 2565 (2021) also expressed “concern that progress in vaccine access has been uneven and *recognizing *that those affected by conflict and insecurity are particularly at risk of being left behind.”^10^ Inadequate health and technology infrastructure, lack of access to water and sanitation, limited government effectiveness, limited access to health and social services, threat to health and care workers, security challenges and misinformation will make COVID-19 vaccination an arduous task in countries facing conflict.

 It has been well documented that the most potent tool to improve immunity and reduce mortality due to COVID-19 is to vaccinate as many individuals and as quickly as possible.^11^ The World Health Organization (WHO) mandates that vaccinating at least 60%-70% of the population to prevent future COVID-19 infection. Since December 2020, countries have accelerated the process of vaccination against COVID-19 through in-country programs, foreign assistance, and multilateral agreements. However, the coverage of vaccination differs drastically between FCS countries and non-FCS countries.^12,13^

 According to People Vaccine Alliance, estimated 9 out of 10 people in low-income countries (LICs) are unlikely to receive a vaccine in 2021.^14^ As on July 2021, high-income countries (HICs) procured 10 times more COVID-19 doses than LICs whereas the population size of HICs is only 2 times more than LICs. To put these statistics into context, HICs have secured, on average, 2 doses per person whereas LICs, on average, have been able to secure half a dose for a person.^15^

 As on July 31, 2021, the COVAX facility procured 2.5 billion doses of COVID-19 vaccine which would be distributed among 138 country participants – many of them are low income and low- and middle-income countries (LMICs) which are also over-represented among fragile, and conflict affected situations. Out of these, slightly more than 215 million doses have been shipped which shows the long road ahead for COVAX’s target countries to vaccinate their residents.^16^

 Determinants of COVID-19 vaccination coverage and implementation of interventions to improve vaccination coverage in FCS and non-FCS should be understood from the contextual differences in social, economic, health system, governance, and political institutions perspective.^12,17^ Identification of the determinants of COVID-19 vaccination coverage in the FCS and non-FCS will assist policy makers to formulate context specific and comprehensive COVID-19 vaccination strategies. This study also contributes to the literature on social model of health. The model highlights the importance of the context, circumstances and surrounding environment that determine health of the individuals and society in the FCS.

###  Conceptual Framework

 Our study substantiates the factors associated with COVID-19 vaccine coverage based on the Council on Social Determinants of Health (CSDH) framework. According to WHO, Social Determinants of Health (SDH) are the non-medical factors that influence health outcomes.^18^ The prejudiced and avoidable differences in health, education, employment, housing, social protection, socioeconomic and socio-political conditions have a major influence on health inequities and public health outcomes. According to the WHO, SDH account for 30%-55% of health outcomes.^19^ To understand health outcomes as a social phenomenon, we require more complex forms of inter-disciplinary and intersectoral policy actions.

 The conceptual framework on CSDH, set up by the WHO, was based on specific theories of the social production of health-psychosocial approaches; social production of disease/political economy of health; and eco-social frameworks.^18^ All three theories use “social selection,” or social mobility, “social causation,” and life course perspectives as the main pathways and mechanisms to explain relationship. According to the CSDH framework, structural features of a society, economy and polity influence the positions and hierarchies prevalent in a populace. These features are rooted in the key institutions and processes of the socioeconomic and political context. For instance, a constitutional democracy would, ideally, have constitutional safeguards for the right to health which ensures equitable accessibility to health services for its people and consequently, a higher vaccine coverage is to be expected.^20,21^ The context and the structures along with social and economic position of the individual determine the SDH inequities in a society. The underlying SDH inequities interact with psychosocial factors, biological factors and health system factors (intermediary determinants) to shape health outcomes.^22^

 FCSs experience different socioeconomic conditions, lack of access to education and health; strained health system; governance and conflict related crisis.^23^ In line with the above, we define the conceptual framework for this study based on an adaptation of the CSDH framework to inequities in COVID-19 vaccine coverage (see Figure). In this model, we posit the SDH on socioeconomic factors, governance and political factors and health system factors which can be observed across a set of countries. These factors individually and jointly influence access, distribution, and overall coverage of COVID-19 vaccines. Importantly, we focus on coverage of vaccines rather than distribution of vaccines.

**Figure:**
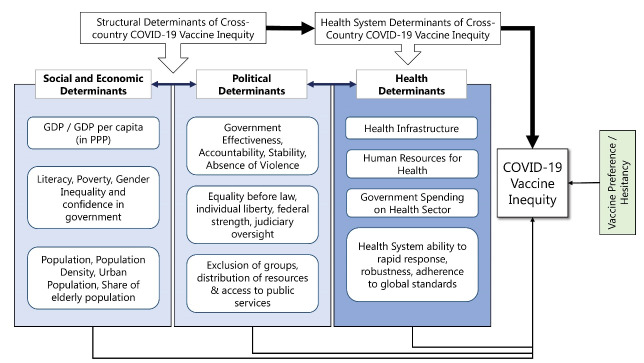
Modified CSDH Framework to Study Cross-country Inequities in COVID-19 Vaccine Coverage. Source: Author’s adaptation of the CSDH framework.^18^ Abbreviations: COVID-19, coronavirus disease 2019; GDP, gross domestic product; CSDH, Council on Social Determinants of Health; PPP, purchasing power parity.

 Evidence suggests that vaccine inequity is led by economic factors which are highly correlated with poverty, literacy, and demographic factors.^24-27^ We view economic and demographic factors reinforcing each other in its effect on vaccine inequity. However, economic growth has empirically been seen to correlate with better governance and democratic credentials – factors which are accounted for by the political dimension of our model.^28,29^ These two factors combine to and shape the health system in a country.^30,31^ Recent literature has also seen the increasing role of vaccine hesitancy in vaccine coverage.^32,33^ We see that as a demand-side factor and incorporate into our model. The modified CSDH model views each dimension as a separate indicator of vaccine inequity as well as views them having a combined effect on vaccine inequity. The study attempts to analyse the strength of each dimension with respect to COVID-19 vaccine inequity.

###  Research Question

 Based on the CSDH framework, we formulate the following research question for this study:

 What are the significant social determinants of COVID-19 vaccination coverage in FCS and non-FCS countries?

## Methods

 Our study analyses data on the COVID-19 pandemic, vaccination coverage and other determinants of vaccination coverage. The indicators were chosen based on availability and authenticity of data, as well as the assumption that they are the best available direct indicators related to the outcomes of the study. All data used in this study were collected from the public databases of - Our World in Data (OWID), WHO, Global Health Security Index (Supplementary file 1), United Nations, World Bank, and other references.

###  Data Management

 This study uses World Bank’s classification of FCS States^34^ as the primary source for country classification (see Table S1 in Supplementary file 1 for a full list of countries). The Financial Year 2022 list classifies 25 countries as conflict affected, 31 countries as fragile and 17 countries as both fragile and conflict for a sample of 39 countries classified as FCS.^34^ These 39 countries have a cumulative population of 930 million residents (12% of the world population). The COVID-19 dataset was retrieved from OWID which includes daily observations on variables such as number of confirmed cases, number of deaths, number of hospital admissions, number of tests and number of people vaccinated from COVID-19.^35^ The OWID dataset includes a sample of 223 countries which is the most representative dataset for COVID-19 related cross-country research. Nevertheless, sampling adequacy for regressions is low due to missing observations for many countries (in both FCS and non-FCS classifications).

 To study the determinants of vaccine coverage, we define the outcome variable as the share of population having received at-least one dose of a COVID-19 vaccine until July 2021.We incorporate perception towards vaccine through data compiled by De Figueiredo et al,^36^ who provide global trends in vaccine confidence across 149 countries. Although the study inquires about vaccine perception to allow for cross-country comparisons, an important caveat is that the author’s gather data on vaccine perception in general and not on COVID-19 vaccine(s) specifically. However, we use the author’s estimate as the best proxy for demand-side behavioural factors influencing COVID-19 vaccine coverage. Interestingly, we observe that confidence in the effectiveness of the vaccine is higher in countries among the FCS than countries in non-FCS.

 The OWID dataset is the master data for this study. Data on socioeconomic variables, health-system variables and political variables have been taken from other reliable sources (see Table S2 in Supplementary file 1 for a full list of variables and their range).

###  Data on Socioeconomic Indicators

 Based on the modified CSDH framework, the study includes gross domestic product (GDP) per capita and an index for socioeconomic resilience as socioeconomic variables. The latter is a composite index of adult literacy rates, United Nations Development Programme’s Gender Inequality Index Score, extreme poverty rates (at purchasing power parity [PPP] $1.90 per capita per day), perceptions on public confidence in the government and strength of domestic media.^37^ We supplement this with population statistics such as population density, share of population above 65 years of age and urban share of population derived from the WHO’s Global Health Repository.^38^

###  Data on Health System Indicators

 Overall health system strength, robustness and responsiveness was assessed from the Global Health Security Index which provides empirically robust indicators from quantitative and qualitative sources on specific health system components. In addition, we use data from WHO’s Global Health Repository to supplement index data with more substantive quantitative data in the form of health infrastructure data, human resources for health data and government health spending data.^37,38^

###  Data on Political Indicators

 We identify two sources of data for political and governance variables, viz. World Bank’s Worldwide Governance Indicators (WGI) and Varieties of Democracy (V-Dem) collection of indices on democracy.^39,40^ The V-Dem project provides a multidimensional view on the complex concept of democracy by identifying five principles of a democracy: electoral, liberal, participatory, deliberative and egalitarian and provides disaggregated data to measure these principles. Primarily, the study uses the WGI variables as they report aggregate and individual governance indicators for 200 countries based on 30 data sources (including V-Dem index). However, (1) due to high degree of correlation between governance and political variables, and (2) absence of societal variables in the WGI, we use indices from the V-Dem collection of indices (see Table S3 in Supplementary file 2).

###  Statistical Methodology

 The study uses STATA v.16.1 IC software to perform its statistical analysis. We use the 5% threshold level of statistical significance for the entire analysis (*P*< .05). This level of significance was chosen owing to the small sample size of countries among the FCS. The outcome variable, ie, vaccine coverage was parametrized using a logarithmic scale. Vaccine coverage was defined as the share of population who received at least one dose COVID-19 vaccine. We conducted a sensitivity analysis for alternate measures of vaccine coverage and found that our results are robust to different definitions of vaccine coverage (see Table S4 in Supplementary file 2). We used multi-variate regression models to assess the relationship between COVID-19 vaccine coverage and cross-country macro-level determinants. The fundamental regression model is as follows:

 ln (*Vax*_i_) = *β*_0_* + β*_1 _*X*_i_* + ϵ*_i_

 Where *Vax*_i_ is the country specific cumulative vaccination coverage at the end of July 2021; *X*_i_ is the vector of independent variables to be possible determinants of vaccine coverage. To account for possible endogeneity, the authors conduct tests for omitted variable bias, linearity, multi-collinearity and heteroskedasticity (Details on the results of these tests can be found in Supplementary file 1). The analysis is conducted on two samples: (1) Non-FCS countries (n=184); (2) FCS countries (as defined by the World Bank) (n=39). Due to the objective of the study to assess and compare the determinants of vaccine coverage, using a binary variable for FCS classification would have yielded an average effect of being in a group on vaccine coverage which is not our intention. Hence, we perform multi-variate regressions on our two samples separately.

## Results

 In absolute terms, countries among FCS have had lower incidence of daily and cumulative confirmed cases and deaths than countries among non-FCS (Table 1). COVID-19 infection waves also coincide within the two groups of countries. The FCS countries have had three peaks in June 2020, January 2021 and April 2021 – coinciding with their respective infection waves.

**Table 1:** Comparison of COVID-19 Caseload, Economic, Demographic, Health Systems, Political Determinants of COVID-19 Vaccine Inequities, by Country Group (Means Compared)

**Indicator**	**Non-FCS**	**FCS**	**Correlation With Vaccine Coverage**
COVID-19 cases, vaccine, perception			
Cumulative cases	192 167 402	5 231 328	10.74%
Cumulative deaths	4123431	88426	10.71%
Average within group CFR	2.49%	3.02%	-10.66%
Vaccination coverage			
At least 1 dose	38.33%	5.46%	
Fully vaccinated	28.30%	2.90%	
Partly vaccinated	10.23%	2.60%	
% Strongly agree that vaccines are effective	58.60%	70.60%	-34.88%
% Strongly disagree that vaccines are effective	2.30%	3.10%	-6.78%
Health-system variables			
GHS score (0-1)	43.28	28.31	54.54%
Hospital beds per 1000	3.31	1.61	32.15%
Medical doctors per 10 000	22.80	6.33	64.95%
Nurses and midwives per 10 000	50.65	16.09	62.67%
Domestic health spending (% of GDP)	3.77%	2.30%	56.37%
Domestic health spending per capita (international $ PPP)	1198.41	105.03	69.31%
Socio-economic variables			
GDP per capita (international $ PPP)	22 554.42	4750.92	70.64%
Human development index (0-100)	76.11	55.63	79.93%
Demographic variables			
Cumulative population (in millions)	6834.34	930.49	-2.64%
Population density (per km^2^)	521.73	140.70	16.08%
Life expectancy at birth	75.09	65.42	71.86%
Population aged 65 and older	0.09	0.04	63.83%
Political and governance variables			
Electoral democracy index (0-1)	0.56	0.36	49.25%
Voice and accountability (-2.5, 2.5)	0.18	-0.74	57.18%
Political stability and absence of violence (-2.5, 2.5)	0.24	-1.15	62.48%
Government effectiveness (-2.5, 2.5)	0.27	-1.20	77.42%
Regulatory quality (-2.5, 2.5)	0.24	-1.07	73.99%
Rule of law (-2.5, 2.5)	0.21	-1.03	73.20%
Control of corruption (-2.5, 2.5)	0.20	-0.93	71.03%

Abbreviations: FCS, fragile and conflict-affected setting; GHS, Global Health Security; COVID-19, coronavirus disease 2019; GDP, gross domestic product; PPP, purchasing power parity; CFR, case fatality rate. Source: OWID dataset, GHS Index, WHO GHO Data Repository, World Bank World Development Indicators, V-Dem Project, World Bank Worldwide Governance Indicators.

 A closer look at the relative measure of mortality – the case fatality rate (CFR) – reveals that in 15 out of 19 months of the pandemic, average CFR among FCS countries was larger or equal to the average CFR in non-FCS countries. The discrepancy in CFR can be attributed to the weak healthcare infrastructure, poverty, informality, lack of access to health services due to conflict, prevalence of comorbidities among other structural reasons.^41,42^ The combination of a high CFR and weak health infrastructure amplify the already existing drivers of food insecurity, lack of employment, gender inequality and social, economic and political instability in FCS countries which in turn can impact health outcomes in FCS countries.^43^

###  Socioeconomic Determinants of Vaccine Coverage

 The combination of economic and demographic factors explains 66% and 48% of the variation in vaccine coverage in non-FCS and FCS countries, respectively. With respect to economic factors, we see that a 1 % increase in GDP per capita is associated with a 0.64% increase in vaccination coverage in non-FCS and a 0.9% increase in FCS countries. Similarly, with a unit increase in the index to measure socioeconomic resilience, vaccine coverage is associated with a 2.7% increase in non-FCS countries and 2.1% increase in FCS countries. On the other hand, demographic factors such as population density, share of older population and size of urban population do not have a strong effect on vaccination coverage. However, population size appears to have a positive influence on vaccine coverage in non-FCS countries and a negative influence in FCS countries. However, demographic factors in total do not appear to be statistically significant drivers of vaccine coverage. In the presence of economic factors, demographic variables lose their significance both within and across the two groups (Refer to Table S5 in Supplementary file 2).

###  Health System Determinants of Vaccine Coverage

 Based on the selection of health system indicators from the Global Health Security Index and WHO’s data on human resources for health and health spending, we see that health systems indicators explain less variation in vaccination coverage than socioeconomic factors in non-FCS countries (R^2^ of 43% for non-FCS countries and 48% for FCS countries). Nevertheless, the major health system related factors appear to be density of human resources for health and government health spending. In non-FCS countries, for every additional doctor per 10 000 population, vaccination coverage is associated with a 1.7% increase and for every nurse or midwife per 10 000 population, vaccination coverage is associated with a 0.04% increase. In FCS countries, density of doctors is negatively associated with vaccination coverage whereas density of nurses and midwives is positively associated. Contrary to the mixed evidence for human resources for health, domestic spending on healthcare has an unambiguous positive effect on vaccination coverage in both non-FCS and FCS countries. For every 1 percentage point increase in health spending as a share of GDP, vaccination coverage is associated with a 13% increase in non-FCS countries and FCS countries are associated with an 8.3% increase (Refer to Table S6 in Supplementary file 3).

###  Political Determinants of Vaccine Coverage

 Under the CSDH framework, structural factors such as freedom of expression, association, political stability and accountability, governance, extent of exclusion of certain groups in the country and executive structure might be seen as strong determinants on a country’s vaccine coverage capacity.

 Our analysis suggests that stronger the government’s capacity to formulate and implement policies, greater its association with vaccine coverage. This effect is stronger in FCS countries than in non-FCS countries. Importantly, the effect of government effectiveness in conflict affected countries appears to be statistically significant indicating that a strong and effective policy implementation apparatus positively contributes to vaccine coverage in conflict affected countries.

 Additionally, countries with greater restrictions/exclusion on access and participation of socioeconomic groups to public spaces are negatively associated with vaccine coverage whereas countries where social groups are excluded from public spaces, vaccine coverage is seen to be higher in such countries which highlight the importance of looking at socioeconomic and societal groups with a separate lens. Although equality before the law and individual liberty is positively correlated with vaccine coverage for all groups of countries, when controlling for the effect of all other independent variables on vaccine coverage, we observe a negative association of equality and liberty with vaccine coverage. Moreover, this result is stronger and statistically significant for FCS countries than for non-FCS countries (Refer to Table S7 in Supplementary file 3).

###  Aggregate Determinants of Vaccine Coverage

 Our last regression model shows estimates from combining socioeconomic, political and health system determinants as possible factors influencing COVID-19 vaccine coverage. Overall, we see that economic factor such as GDP per capita and socioeconomic resilience play the strongest role in influencing vaccine coverage (Table 2). However, where GDP per capita is a stronger factor for non-FCS countries, socioeconomic factors such literacy, poverty levels and gender equality play a much larger role in FCS countries. Size of the country in terms of its population has a significant positive effect in vaccine coverage for non-FCS countries whereas FCS countries with larger population sizes are associated with lesser vaccine coverage, *ceterus paribus*. Effective government, more power to regional governments, political stability and absence of violence plays a much larger role for FCS countries than non-FCS countries judging by the magnitude of their relative coefficients with vaccine coverage. Importantly, when controlling for other factors, equality before the law and individual liberty has a negative association with vaccine coverage indicating an avenue for further research. Regarding health systems, we find inconclusive evidence regarding the role of human resources for health in influencing vaccine coverage. Moving from the structural factors to behavioural factors, we proxy for vaccine hesitancy by a variable from De Figueiredo et al^36^ which measures preference towards pre-COVID-19 vaccine effectiveness. We see those non-FCS countries with a higher share of people strongly believing in the effectiveness of a vaccine are associated with lower rates of COVID-19 vaccine coverage, *ceterus paribus*. On the other hand, FCS countries with a higher share of people strongly believing in the vaccine effectiveness have a positive association with COVID-19 vaccine coverage. With respect to the R^2^, we observe that our set of independent variables explain 68% and 62% of the variation in the vaccine coverage across non-FCS and FCS countries, respectively. Based on the previous discussion where SDH explain on average 30%-55% of the variation in a health metric, our study performs well in explaining variation in the coverage of COVID-19 vaccines.

**Table 2:** Aggregate Determinants of COVID-19 Vaccine Coverage

	**Non-FCS**	**FCS**
Log of GDP per capita	0.49^a^(2.55)	0.28 (0.29)
Socioeconomic resilience	0.02^b^(3.00)	0.05 (1.12)
Population in million	0.00^b^(2.98)	- 0.00 (-0.04)
Government effectiveness	-0.03 (-0.12)	1.39 (0.60)
Index of equality and liberty	-0.05 (-0.28)	-0.89 (-0.59)
Index of regional government power	0.08 (1.25)	-0.17 (-0.19)
Political stability and absence of violence	0.17 (0.91)	0.27 (0.32)
Density of doctors per 10 000 population	0.00 (1.66)	0.04 (0.39)
Density of nurses and midwives per 10 000 population	-0.00 (-1.50)	-0.01 (-0.16)
Domestic government health expenditure (% of GDP)	0.047 (0.77)	-0.164 (-0.28)
Share of population strongly agrees that vaccines are effective	-0.00 (-0.55)	0.07 (1.44)
Constant	-3.41^c^(-1.91)	-7.57 (-1.07)
*R*^2^	0.68	0.63
Observations	103	19

Abbreviations: FCS, fragile and conflict-affected setting; COVID-19, coronavirus disease 2019; GDP, gross domestic product. *t* statistics in parentheses. Heteroskedasticity robust standard errors, tested for multi-collinearity, linearity and model specification. ^a^ *P* <.05, ^b^*P* <.01, ^c^*P* <.10.

## Discussion

 Leaders of the G20 and other states, at the Global Health Summit in Rome reiterated that the pandemic remains to be the public health emergencies of international concern with its impact on the most vulnerable sections of the society.^44^ Principles of the Rome declaration underscore the efforts needed to enhance timely, global and equitable access to safe, effective and affordable COVID-19 tools. The Rome declaration further recognizes the role of extensive COVID-19 vaccination as a global public good, by extending support to Access to COVID-19 Tools Accelerator.^45^ Even though the declaration articulates the need for effective governance, multi-lateral cooperation, and promotion of people centric, sustainable and evidence-based policies there is a noticeable gap in the burden of diseases, vaccination coverage and implementation of COVID-19 prevention and control policies globally and more so in the FCSs. In this context, this study has explored to map the determinants of COVID-19 vaccination coverage in FCS and non-FCS countries. Principles articulated in the Rome declaration are implementable provided the context is conducive for the implementation because the gaps in pandemic preparedness were already existent pre-COVID-19. Social position of an individual determines the vulnerability to various disease conditions and health outcomes.^46^

 Our results highlight the role of economic, political, and health system factors along with vaccine hesitancy in influencing COVID-19 vaccine coverage. Vaccine hesitancy is a complex phenomenon which is influenced by religion, low perceived risk of the disease, lack of transparency in the vaccine development and misinformation surrounding these vaccines.^47^ A study by Dahie et al, emphasized the need for addressing vaccine hesitancy among Somalians to improve COVID-19 vaccine coverage.^48^ Similarly, a review focusing on LMICs concludes that there is an immediate need to address vaccine hesitancy by building^49^ “public acceptability, trust and concern over the safety and benefit of approved vaccines,” through evidence-based health communication and advisory.^50^

 We see that socioeconomic factors such as GDP per capita and socioeconomic resilience are associated with the highest vaccination coverage for COVID-19 in both non-FCS and FCS. A study by Zhu et al stressed on the importance of reducing socioeconomic inequalities and strengthening the resilience of health systems to better respond to public health emergencies globally.^51^

 Evidence further emphasized on the significant role played by health expenditures per capita, governments involvement in health expenditures, GDP per capita, and industry share in GDP.^41^ Similar study done in SAARC-ASEAN (South Asian Association for Regional Cooperation-Association of Southeast Asian Nations) region highlighted that health expenditure in the SAARC-ASEAN region should be increased as results indicated that it improved the health status of the population in the region.^52^

 From the empirical evidence we conclude that political factors are important determinants of COVID-19 vaccine coverage. Due to the relative paucity in empirical literature exploring the linkages between political factors and vaccine coverage on a cross-country basis, we provide the prima-facie evidence regarding these linkages. We conclude that government effectiveness is the strongest and the most statistically significant factor in increasing vaccine coverage, other factors such as role of regional governments, equality and individual liberty, exclusion of socioeconomic and social groups and political stability are strong factors influencing vaccine coverage. A study by Murtin et al shows that both government competence and values are strong predictors of public trust.^53^ The success of vaccination campaigns will largely be influenced by the extent to which people trust the effectiveness and safety of the vaccines, the competence and reliability of the institutions that deliver them, and the principles that guide government decisions and actions. Further research must be done in exploring the linkages of more structural factors such as equality and liberty, exclusion of certain groups, voice and accountability in the access and distribution of the COVID-19 vaccine.

 FCSs and LMIC experienced unprecedented consequences on patients, caretakers, healthcare providers, building blocks of health systems, and financial systems. In LMIC and FCS settings, COVID-19 effects could be even more severe due to lack of preparedness and response plans to the pandemic. Shortage of healthcare providers, infrastructure, and limited health budget impacts pandemic prevention and control measures.^54,55^

 For health systems, we conclude that human resources for health and domestic health spending are the major factors influencing vaccination coverage. Factors such as hospital beds density, measles immunization coverage, and other indices to measure overall health system’s ability to respond to health crisis do not appear to influence COVID-19 vaccination coverage.^56^ On the contrary, we see idiosyncratic evidence arguing that non-FCS countries adhering to global health norms are negatively associated with vaccine coverage highlighting the role of further research to investigate the complexities of health systems and its relationship with COVID-19 vaccine coverage.

 The study has various strengths. We have attempted to understand the SDH by including variables beyond the health sector. We have also laid emphasis on the FCSs and have identified the right determinants which will aid in designing interventions in the FCS, thereby making this a novel approach. We have adopted a modified CSDH framework to include socioeconomic, governance, political fand health system factors which often interplay to influence vaccine coverage for the COVID-19. There is limited literature on the COVID-19 vaccination coverage in FCS and we felt the moral obligation to unearth the role of various determinants that affect vaccine coverage in these settings.^57^ We have therefore contributed to the empirical evidence towards COVID-19 vaccination in FCS settings. We have also employed methods that are easily replicable and are statistically sound in identifying relationships between outcome variable and determinants.

 Our study has certain limitations such as the analysis is based on country averages and the data were merged from multiple sources. The number of countries classified as FCS are small, therefore we should draw inferences with caution. Coefficients are sensitive to the date of research and results of the study are sensitive to more data.

## Conclusion

 The study has stimulated a thinking on the multifactorial influence of various determinants on vaccination coverage in FCS and non-FCS. Furthermore, the study confirmed that political factors, government effectiveness and political stability are also important determinants of vaccine coverage. The result of this study draws attention to few policy implications such as promoting future research to explore the linkages between the perceived equality before the law and individual liberty and its effect on vaccination coverage in the FCS. Secondly, the pandemic is far from over until we adopt and implement inclusive global policies that can effectively address vaccine famines in FCS.

## Ethical issues

 Ethics approval was not taken as the data was open access and in the public domain.

## Competing interests

 Authors declare that they have no competing interests.

## Authors’ contributions

 Conception and design: SP, MP, and NG. Acquisition of data: MP, SP, and NG. Analysis and interpretation of data: MP, SP, and NG. Drafting of the manuscript: SP, MP, and NG. Critical revision of the manuscript for important intellectual content: SP, NG. Statistical analysis: MP. Administrative, technical, or material support: SP, MP, and NG. Supervision: SP.

## Supplementary files

Supplementary file 1 contains Tables S1 and S2.

Supplementary file 2 contains Tables S3-S5.

Supplementary file 3 contains Tables S6 and S7.
