# Supplementary material for: A Comparative Analysis on the Social Determinants of COVID-19 Vaccination Coverage in Fragile and Conflict Affected Settings and Non-fragile and Conflict Affected Settings
Source: Int J Health Policy Manag. 2022 Oct 10;12:6830. doi: 10.34172/ijhpm.2022.6830 (PMC10125044; doi:10.34172/ijhpm.2022.6830)
Supplement: Supplementary file 1 — contains Tables S1 and S2. [file ijhpm-12-6830-s001.pdf]

Article title: A Comparative Analysis on the Social Determinants of COVID-19 Vaccination Coverage in Fragile and Conflict Affected Settings and Non-fragile and Conflict Affected Settings

Journal name: International Journal of Health Policy and Management (IJHPM)

Authors' information: Sanjay Pattanshetty[1]*, Mantej Pardesi[2], Nachiket Gudi[3]

[1]Department of Global Health Governance, Prasanna School of Public Health, Manipal Academy of Higher Education, Manipal, India.

[2]Department of Microeconomics and Public Economics, School of Business and Economics, Maastricht University, Maastricht, The Netherlands.

[3]Public Health Evidence South Asia, Department of Health Information, Prasanna School of Public Health, Manipal Academy of Higher Education, Manipal, India.

(*Corresponding author: sanjay.pattanshetty@manipal.edu)

Supplementary file 1

I. Global Health Security Index

The Global Health Security Index (GHS) is an assessment of global health security capabilities in 195 countries prepared by the John Hopkins Center for Health Security, Nuclear Threat Initiative and The Economist's Intelligence Unit. The index is based on a questionnaire of 140 questions, organized across 6 categories, 34 indicators, and 85 sub indicators[1]. The six categories are:

- **Prevention:** Prevention of the emergence or release of pathogens.
- **Detection and Reporting:** Early detection and reporting for epidemics of potential international concern.
- **Rapid Response:** Rapid response to and mitigation of the spread of an epidemic
- **Health System:** Sufficient and robust health system to treat the sick and protect health workers.
- **Compliance with International Norms:** Commitments to improving national capacity, financing plans to address gaps, and adhering to global norms.
- **Risk Environment:** Overall risk environment and country vulnerability to biological threats.

The index relies entirely on open-source information. The researchers worked with an international advisory panel of 21 experts from 13 countries.

The top 10 best performing countries on this index are United States, United Kingdom, Netherlands, Australia, Canada, Thailand, Sweden, Denmark, South Korea and Finland. The bottom 10 worst performing countries on this index are Equatorial Guinea, Somalia, North Korea, Sao Tome and Principe, Marshall Islands, Yemen, Kiribati, Syria, Guinea-Bissau, Gabon and the Cook Islands.

II. World Bank FCS Classification

Following is the list of countries that have been listed as fragile and conflict affected as per the World Bank's FCS classification.

Table S1: *List of countries identified as Fragile and Conflict Affected, by type of classification*

List of Countries	FCS-WB	Conflict Affected	Fragile	List of Countries	FCS-WB	Conflict Affected	Fragile
Afghanistan	✓	✓	✓	Lebanon	✓		✓
Armenia	✓	✓		Libya	✓	✓	✓
Azerbaijan	✓	✓		Mali	✓	✓	
Burkina Faso	✓	✓		Marshall Islands	✓		✓
Burundi	✓	✓	✓	Micronesia (country)	✓		✓
Cameroon	✓	✓	✓	Mozambique	✓	✓	
Central African Republic	✓	✓	✓	Myanmar	✓	✓	
Chad	✓	✓	✓	Niger	✓	✓	
Comoros	✓		✓	Nigeria	✓	✓	✓
Congo	✓		✓	Palestine	✓	✓	✓
Democratic Republic of Congo	✓	✓	✓	Papua New Guinea	✓		✓
Eritrea	✓		✓	Solomon Islands	✓		✓

Ethiopia	✓	✓	✓	Somalia	✓	✓	✓
Guinea-Bissau	✓		✓	South Sudan	✓	✓	✓
Haiti	✓	✓	✓	Sudan	✓	✓	✓
Iraq	✓	✓		Syria	✓	✓	✓
Kiribati	✓		✓	Timor	✓		✓
Kosovo	✓		✓	Tuvalu	✓		✓
				Venezuela	✓	✓	✓
				Yemen	✓	✓	✓
				Zimbabwe	✓		✓

Source: World Bank FCS Classification, FSI index, ACLED Data

The World Bank classified FCS[2] countries based on the nature and severity of the issues they face. These include countries with high levels of institutional and social fragility and countries affected by violent conflict. The World Bank defines fragile states according to their ranking in the Country Policy and Institutional Assessment (CPIA) that includes a set of 16 criteria grouped in four clusters: economic management, structural policies, policies for social inclusion and equity, and public sector management and institutions. Fragile Situations include countries or territories with (i) a harmonized CPIA country rating of 3.2 or less, and/or (ii) the presence of a UN and/or regional peacekeeping or political/peace-building mission during the last three years. Conflict is measured using thresholds on conflict related deaths synchronised between the Armed Conflict Location and Event Data Project (ACLED)[3] and the Uppsala Conflict Data Program (UCDP)[4] data. We further divide the aggregate category of FCS into two separate categories for Fragile countries and Conflict affected countries. A country can be in either of the two or in both the categories. In total we have 39 countries which are classified as Fragile and Conflict affected out of which 25 are conflict affected, 31 are institutionally fragile and 17 are both fragile and conflict affected. Due to data limitations, sampled observations for multivariate regressions is considerably lower than the full sample of FCS countries. Therefore, our results must be interpreted with caution. A more detailed analysis should follow our approach where efforts are made to compare similar FCS and non-FCS countries. In such a case, deviation in vaccine coverage would be explained by unobserved social determinants.

III. List of variables used in the analysis

The study uses data from various sources as described in the following table.

Table S2: Description of variables

Type of variable	Name of variable	Source
**COVID-19 related variables**	Cases, Deaths, Tests undertaken, Stringency index, Vaccination Coverage, Hospital Beds per thousand residents	Our World in Data[5]
**Economic variables**	GDP per capita and Human Development Index	World Bank- World Development Indicators[6]
**Demographic variables**	Population size, population density, population aged 65 years and older, Life expectancy	Our World In Data[5]
**Health System variables**	Global Health Security Index	Global Health Security Index[1]
	Measles immunization coverage, Density of Doctors, Nurses and Midwives per 10000 population, Domestic government health expenditure (as a % of GDP and as per capita PPP $)	WHO Global Health Observatory Data Repository[7]
**Governance Indicators**	Worldwide Governance Indicators (Voice and Accountability, Political Stability and Absence of Violence, Rule of Law, Regulatory Quality, Control of Corruption, Government Effectiveness)	World Bank[8]
**Political variables**	Indices for electoral democracy, equality and liberty, regional government power, judicial constraints on the executive, equal distribution of resources, exclusion of social and socio-economic groups	V-Dem project[9]
