# Supplementary material for: A Comparative Analysis on the Social Determinants of COVID-19 Vaccination Coverage in Fragile and Conflict Affected Settings and Non-fragile and Conflict Affected Settings
Source: Int J Health Policy Manag. 2022 Oct 10;12:6830. doi: 10.34172/ijhpm.2022.6830 (PMC10125044; doi:10.34172/ijhpm.2022.6830)
Supplement: Supplementary file 2 — contains Tables S3-S5. [file ijhpm-12-6830-s002.pdf]

Article title: A Comparative Analysis on the Social Determinants of COVID-19 Vaccination Coverage in Fragile and Conflict Affected Settings and Non-fragile and Conflict Affected Settings

Journal name: International Journal of Health Policy and Management (IJHPM)

Authors' information: Sanjay Pattanshetty[1]*, Mantej Pardesi[2], Nachiket Gudi[3]

[1]Department of Global Health Governance, Prasanna School of Public Health, Manipal Academy of Higher Education, Manipal, India.

[2]Department of Microeconomics and Public Economics, School of Business and Economics, Maastricht University, Maastricht, The Netherlands.

[3]Public Health Evidence South Asia, Department of Health Information, Prasanna School of Public Health, Manipal Academy of Higher Education, Manipal, India.

(*Corresponding author: sanjay.pattanshetty@manipal.edu)

Supplementary file 2

To complement Table 1, following are the detailed definitions of key dependent variables used in our analysis:

Table S3: Definitions of key dependent variables used

1.	**Log of GDP Per Capita**	Natural Logarithm of Gross domestic product at purchasing power parity (constant 2011 international dollars), most recent year available. [10]
2.	**Index for Socio-economic Resilience**	A composite sub-index of GHS Index scoring countries on their economic health. This index includes literacy rates, categorical measures for gender equality, social inclusion, public confidence in government, inequality and media. Range: [15,100]<br><br>For more information, please refer to: https://www.ghsindex.org/wp-

		content/uploads/2021/11/2021_GHSindex_Methodology_FINAL.pdf
3.	**Population Density**<br><br>**Aged 65 and Older**<br><br>**Population by million**<br><br>**Urban Share of Residents**	Number of people divided by land area, measured in square kilometers, most recent year available. [10]<br><br>Share of the population that is 65 years and older, most recent year available. [10]<br><br>Population divided by 1,000,000. [10]<br><br>Share of the population living in urban areas, most recent year available.[10]
4.	**Hospital beds per thousand residents**<br><br>**Medical doctors per 10000 population**<br><br>**Nurses/Midwives per 10000 population**	Hospital beds per 1,000 people, most recent year available since 2010.[10]<br><br>Number of medical doctors for every 10000 population.[7]<br><br>Number of nurses and midwives for every 10000 population.[7]
5.	**Index for voice and accountability**	Aggregate index to capture perceptions of the extent to which a country's citizens are able to participate in selecting their government, as well as freedom of expression, freedom of association, and a free media.[8]
6.	**Index for political stability**	Aggregate index to capture the likelihood of political instability and/or politically motivated violence, including terrorism.[8]
7.	**Index for Government effectiveness**	Aggregate index to capture perceptions of the quality of public services, the quality of the civil service and the degree of its independence from political pressures, the quality of policy formulation and implementation, and the credibility of the government's commitment to such policies.[8]

8.	**Index for exclusion of economic groups**	Index for political exclusion of socio-economic groups. “Exclusion” is when individuals are denied access to services or participation in governed (public) spaces based on their identity or belonging to a particular group.[9]
9.	**Index for exclusion of social groups**	Index for exclusion of social groups. “Exclusion” is when individuals are denied access to services or participation in governed spaces based on their identity or belonging to a particular group.[9]
10	**Index for equal distribution of resources**	Index to measure the extent to which resources (both tangible and intangible) are distributed in the society.[9]
11	**Index for the power of regional government**	Index to measure the strength of elected regional governments. A high score is given to countries who have elected regional government that are able to operate without restrictions from unelected actors at the regional level with the exception of judicial bodies.[9]
12	**Index for equality and liberty**	Index to assess the extent of transparency and enforcement of the public administration, extent to which citizens enjoy access to justice, secure property rights, freedom from forced labour, freedom of movement, physical integrity rights and freedom of religion.[9]
13	**Index for judicial constraints**	Index to assess the extent with which the executive respect the constitution and comply with court rulings, and to what extent is the judiciary able to act in an independent fashion.[9]

IV. Sensitivity Analysis of outcome variable

To test for sensitivity in out outcome variable, we test the regressions based on three alternate definitions of vaccine coverage, i.e., vaccine coverage as doses administered, as share of population fully vaccinated and as the average daily vaccination rate. **Error! Reference source not found.** shows the regressions for four outcome variables. We observe that each of the four-outcome variable have similar results in terms of magnitude and statistical significance implying our findings are robust to alternate definitions of vaccine coverage. The statistical power of each of the four regressions exceeds 0.65. This mean that, on average, our model captures at-least 65% of the variation in vaccination coverage.

With respect to the variables, we observe strong and statistically significant association of daily vaccinations with political stability, and density of doctors as the only unique result compared to the other three outcome variables. However, we do not use daily vaccination as a measure for vaccination due to its high correlation with population size.

Table S4: Sensitivity analysis of determinants to alternate definitions of COVID-19 vaccine coverage

Variable	Vax-Atleast1Dose	Doses Admin	Full Vax	Daily Vax
**Log of GDP per capita**	0.52^{**}<br>(3.21)	0.57^{***}<br>(3.42)	0.84^{***}<br>(3.70)	0.57^{***}<br>(4.27)
**Socio-economic resilience**	0.03^{**}<br>(3.34)	0.03^{**}<br>(3.24)	0.03^{**}<br>(2.70)	0.02^{**}<br>(3.22)
**Population in million**	0.00^{*}<br>(2.50)	0.00^{***}<br>(3.39)	0.00<br>(1.04)	0.00^{**}<br>(2.71)
**Index for government effectiveness**	0.05<br>(0.21)	0.05<br>(0.18)	0.00<br>(0.00)	-0.21<br>(-1.11)
**Index of equality and liberty**	-0.15<br>(-0.93)	-0.15<br>(-0.90)	0.02<br>(0.10)	-0.24<br>(-1.81)
**Index of regional government power**	0.07<br>(0.94)	0.04<br>(0.51)	0.01<br>(0.13)	0.00<br>(0.04)
**Index for political stability and absence of violence**	0.36^{*}<br>(2.00)	0.38<br>(1.98)	0.32<br>(1.40)	0.54^{***}<br>(3.87)
**Density of doctors**	0.01<br>(1.69)	0.01<br>(1.72)	0.01<br>(1.47)	0.01^{*}<br>(2.18)
**Density of nurses and**	-0.01	-0.00	-0.01	-0.00

**midwives**	(-1.91)	(-1.67)	(-1.53)	(-1.36)
**Domestic Govt. Health Exp (% of GDP)**	0.04<br>(0.65)	0.03<br>(0.58)	0.03<br>(0.43)	0.03<br>(0.58)
**Share of population strongly agrees that vaccines are effective**	-0.00<br>(-0.15)	-0.00<br>(-0.44)	-0.00<br>(-0.49)	0.00<br>(0.42)
**Constant**	-4.19** (-2.90)	-4.19** (-2.76)	-7.72*** (-3.47)	0.06<br>(0.05)
R^2	0.71	0.72	0.68	0.75
**Observations**	122	122	119	121

t statistics in parentheses

* $p < 0.05$, ** $p < 0.01$, *** $p < 0.001$

Coefficients rounded off to two digit decimal places

V. Regression Analysis: Socio-economic determinants of COVID-19 vaccine coverage

Table S5: Socio-economic determinants of COVID-19 vaccine coverage

Variable	Non-FCS	FCS-WB
**Log of GDP per capita**	0.64*** (5.79)	0.90 (1.64)
**Socio-economic resilience**	0.03*** (4.34)	0.02 (0.69)
**Population Density per square kilometer**	0.00 (0.33)	0.00 (0.39)
**Share of population 65 and above**	0.00 (0.35)	0.01 (0.09)
**Population in million**	0.00** (2.80)	-0.01 (-1.03)
**Percentage of urban population in 2020**	-0.01 (-1.28)	-0.01 (-0.65)
**Constant**	-4.72*** (-6.78)	-7.20* (-2.36)
R^2	0.66	0.48
**Observations**	144	27

t statistics in parentheses

Heteroscedasticity robust standard errors, tested for multi-collinearity, linearity and model specification

$^{+}$ $p < 0.10$, * $p < 0.05$, ** $p < 0.01$, *** $p < 0.001$
Coefficients rounded off to two digit decimal places
