# Supplementary material for: A Comparative Analysis on the Social Determinants of COVID-19 Vaccination Coverage in Fragile and Conflict Affected Settings and Non-fragile and Conflict Affected Settings
Source: Int J Health Policy Manag. 2022 Oct 10;12:6830. doi: 10.34172/ijhpm.2022.6830 (PMC10125044; doi:10.34172/ijhpm.2022.6830)
Supplement: Supplementary file 3 — contains Tables S6 and S7. [file ijhpm-12-6830-s003.pdf]

Article title: A Comparative Analysis on the Social Determinants of COVID-19 Vaccination Coverage in Fragile and Conflict Affected Settings and Non-fragile and Conflict Affected Settings

Journal name: International Journal of Health Policy and Management (IJHPM)

Authors' information: Sanjay Pattanshetty[1]*, Mantej Pardesi[2], Nachiket Gudi[3]

[1]Department of Global Health Governance, Prasanna School of Public Health, Manipal Academy of Higher Education, Manipal, India.

[2]Department of Microeconomics and Public Economics, School of Business and Economics, Maastricht University, Maastricht, The Netherlands.

[3]Public Health Evidence South Asia, Department of Health Information, Prasanna School of Public Health, Manipal Academy of Higher Education, Manipal, India.

(*Corresponding author: sanjay.pattanshetty@manipal.edu)

Supplementary file 3

VI. Regression Analysis: Health System determinants of COVID-19 vaccine coverage

Table S6. Health System determinants of COVID-19 vaccine coverage

Variable	Non-FCS	FCS-WB
**Hospital Beds per Thousand residents**	-0.05<br>(-1.13)	0.91<br>(1.00)
**Density of Doctors (per 10000 population)**	0.02**<br>(2.87)	-0.07<br>(-0.56)
**Density of Nurses and Midwives (per 10000 population)**	0.00*<br>(2.02)	0.03<br>(0.59)
**Measles Immunization Coverage**	0.01<br>(1.55)	0.00<br>(0.11)
**Domestic Govt. Health Exp (% of GDP)**	0.14**<br>(2.74)	0.08<br>(0.31)
**Health system rapid response ability**	0.01<br>(0.94)	-0.01<br>(-0.26)

**Health system robustness**	0.01<br>(0.68)	0.08<br>(0.79)
**Health system commitment to global norms**	-0.02*<br>(-2.27)	0.01<br>(0.13)
**Constant**	1.35^{+}<br>(1.90)	-1.92<br>(-0.60)
R^2	0.44	0.49
**Observations**	126	18

t statistics in parentheses

Heteroscedasticity robust standard errors, tested for multi-collinearity, linearity and model specification

$^{+}$ $p < 0.10$, * $p < 0.05$, ** $p < 0.01$, *** $p < 0.001$
Coefficients rounded off to two digit decimal places

VII. Regression Analysis: Political Determinants of COVID-19 Vaccine Coverage

Table S7: Regression Analysis: Political determinants of COVID-19 Vaccine Coverage

Variable	Non-FCS	FCS-WB
**Voice and Accountability**	0.23<br>(0.99)	1.06<br>(0.85)
**Political Stability**	0.19<br>(1.16)	0.60<br>(1.45)
**Government Effectiveness**	0.69***<br>(4.13)	0.85<br>(1.03)
**Exclusion of social groups**	-0.51^{+}<br>(-1.70)	-0.72<br>(-1.37)
**Exclusion of socio-economic groups**	0.42*<br>(2.01)	0.27<br>(0.50)
**Equal distribution of resources**	0.07<br>(0.34)	0.56<br>(1.16)
**Regional Government Power**	0.23**<br>(3.16)	-0.02<br>(-0.07)
**Judicial constraints on the executive**	0.05<br>(0.31)	-0.03<br>(-0.05)
**Equality and Liberty**	-0.34<br>(-1.46)	-1.64**<br>(-2.85)
**Constant**	2.80***<br>(27.52)	3.10***<br>(4.79)
R^2	0.52	0.41
**Observations**	135	33

t statistics in parentheses

Heteroscedasticity robust standard errors, tested for multi-collinearity, linearity and model specification

$^{+}$ $p < 0.10$, * $p < 0.05$, ** $p < 0.01$, *** $p < 0.001$
Coefficients rounded off to two digit decimal places

VIII: Diagnostic tests for omitted variable bias, multi-collinearity, linearity and heteroscedasticity.

For each level of analysis, i.e., socio-economic determinants, political determinants, health-system determinants, we perform a series of diagnostic tests to check for endogeneity.

1. Model specification

We perform a link test to assess model specification. This test is based on the idea that if a regression is properly specified, one should not find additional independent variables that are significant (except by chance). This means that the dependent variable needs a transformation or "link" function to relate to the independent variables. Operationally, we add an independent variable to the equation that is likely to be significant if there is a specification error. For our aggregate analysis, we expect the link test to show misspecification as we only focus on social determinants of health. On one hand, our narrow focus allows us to clearly see specific relations between regressors and vaccine coverage, but on the other hand, leaves scope for further additions to our framework. Consequently, we find that our link test provides evidence of model misspecification. Specifically, when we regress our dependent variable on its predicted values and the square of predictions, we find the squared term to be statistically significant. This means that we can either change the link function (to a generalised model as by Zhu et al.,) or we change measurement of individual variables[11].

2. Omitted Variable Bias

We perform Ramsey's (1969) regression specification error-test for omitted variables on each of our regressions under the null hypothesis that the model has no omitted variables. For the aggregate analysis, we conclude that there is scope for omitted variable bias which is what we expected based on the link test and Ramsey's test.

3. Collinearity

To detect the collinearity of the regressors with the constant, we compute variance inflation factors (VIF) for each of our regressors. Multi-collinearity is seen if, a) the largest VIF is greater than 10, and b) the mean of all VIFs is considerably larger than 1. For the aggregate analysis, we do not find evidence of multi-collinearity as we do not find any regressor to have variance inflation factor greater than 10 and the mean of all VIF's is around 3.02 which, albeit far from 1, can be accommodated in our analysis as insufficient evidence for multi-collinearity.

4. Heteroscedasticity

We employ White's test to check for heteroscedasticity under the null hypothesis that there is no heteroscedasticity with the test statistic having a chi-square distribution with K*(K+3)/2

degrees of freedom (K = number of independent parameters). For our aggregate analysis, we find that p-value for the chi-squared test-statistics to be 0.59 which is greater than 0.05. Consequently, we fail to reject the null hypothesis and can conclude that we have limited the extent of heteroscedasticity in our regression specification. To substantiate this, we also use Breusch-Pagan test for heteroscedasticity (by lifting the normality assumption of error terms) and find that p-value (p=0.046) for the test-statistic (Chi-squared = 19.95) is statistically insignificant at 1% level of significance. The null hypothesis in the Breusch-Pagan test is of constant variance among the independent variables. By failing to reject the null hypothesis, we again conclude that we account for heteroscedasticity in our regression model.

References

1. Nuclear Threat Initiative, Johns Hopkins Center for Health Security, Unit TEI. 2019 Global Health Security Index. 2020. https://www.ghsindex.org/wp-content/uploads/2020/04/2019-Global-Health-Security-Index.pdf .

2. World Bank. *Classification of Fragile and Conflict-Affected Situations*. The World Bank Group; 2021. https://www.worldbank.org/en/topic/fragilityconflictviolence/brief/harmonized-list-of-fragile-situations .

3. ACLED. ACLED. https://acleddata.com/ . Accessed June 7, 2022.

4. UCDP - Uppsala Conflict Data Program. https://ucdp.uu.se/ . Accessed June 7, 2022.

5. Ritchie H, Mathieu E, Rodés-Guirao L, et al. Coronavirus Pandemic (COVID-19). *Our World Data*. March 2020. https://ourworldindata.org/coronavirus . Accessed June 7, 2022.

6. World Development Indicators | DataBank. https://databank.worldbank.org/source/world-development-indicators. Accessed June 7, 2022 .

7. World Health Organization. Global Health Observatory Data Repository. July 2021. https://www.who.int/data/gho .

8. World Bank. Worldwide Governance Indicators. Kaufmann D, Kraay A, eds. *WGI*. 2021. http://info.worldbank.org/governance/wgi/ . Accessed July 31, 2021.

9. Coppedge M, Gerring J, Knutsen CH, et al. V-Dem Dataset v11. 1. 2021.

10. Ritchie H, Mathieu E, Rodés-Guirao L, et al. Coronavirus pandemic (COVID-19). *Our World Data*. 2020.

11. Zhu J, Yan W, Zhu L, Liu J. COVID-19 pandemic in BRICS countries and its association with socio-economic and demographic characteristics, health vulnerability, resources, and policy response. *Infect Poverty*. 2021;10(1):97. doi:10.1186/s40249-021-00881-w
